# Is adiponectin involved in morphea pathogenesis? – first observational study

**DOI:** 10.3389/fimmu.2025.1588439

**Published:** 2025-05-30

**Authors:** Adriana Polańska, Aleksandra Wiktoria Bratborska, Michał J. Kowalczyk, Ryszard Żaba, Aleksandra Dańczak-Pazdrowska

**Affiliations:** ^1^ Department of Dermatology and Venereology, Poznan University of Medical Sciences, Poznan, Poland; ^2^ Doctoral School, Poznan University of Medical Sciences, Poznan, Poland; ^3^ Department of Dermatology, Poznan University of Medical Sciences, Poznan, Poland

**Keywords:** morphea, localized scleroderma, adiponectin, skin fibrosis, skin sclerosis, connective tissue disease, fibrosis biomarkers, fibroproliferative diseases

## Abstract

**Background:**

Morphea is a chronic inflammatory condition characterized by fibrosis of the skin and/or subcutaneous tissues. Adiponectin is an adipokine known for its anti-inflammatory and antifibrotic properties. Lower levels of this protein have been associated with various diseases, but to date, no studies have evaluated adiponectin levels in patients with morphea.

**Aim:**

The purpose of this study was to analyze the serum concentration of adiponectin in patients suffering from different types of morphea. Additionally, we aimed to investigate the relationship between adiponectin levels and clinical parameters, as well as the severity of skin involvement.

**Methods:**

The study involved 67 patients with morphea and 30 healthy controls. Participants from the study group underwent a thorough clinical evaluation. Serum adiponectin levels were measured in both groups using enzyme-linked immunosorbent assay kits (ELISA).

**Results:**

Serum adiponectin concentrations were significantly reduced in morphea patients compared to healthy controls. We observed no significant differences in adiponectin concentrations among the various morphea types; however, patients diagnosed with morphea en plaque (MEP) or generalized morphea (GM) had significantly lower serum adiponectin concentrations compared to healthy subjects. Furthermore, patients presenting with severe forms of the disease [the group included GM, deep morphea (DM), and linear morphea (LM)] had significantly reduced levels of adiponectin compared to healthy subjects. We found no significant differences in adiponectin levels between patients with active disease and patients in the non-active phase. There were no correlations between adiponectin levels and the localized scleroderma assessment tool (LoSCAT) score or disease duration.

**Conclusion:**

Patients with morphea exhibit significantly lower levels of serum adiponectin, yet these levels do not correlate with the disease severity or activity. Further research is needed to explore the potential role of adiponectin in the pathogenesis of morphea.

## 1 Introduction

Morphea, also known as localized scleroderma, is a chronic inflammatory condition characterized by fibrosis and sclerosis in the skin and/or subcutaneous tissues. In contrast to systemic sclerosis (SSc), morphea does not involve internal organs and is confined to the dermal and subcutaneous layers, although it may, in rare instances, extend to the underlying structures such as fascia, muscles, or bones. The pathogenesis of morphea involves a complex interplay between genetic susceptibility, environmental factors, and immune dysregulation. Crucial to initiating the fibrotic process is the activation of the immune system, which includes T-cell-mediated responses and the production of autoantibodies. The activated T cells release proinflammatory interleukin (IL)-1β, IL-4, IL-6, IL-10, IL-17A, IL-27, as well as transforming growth factor β (TGF-β) and interferon γ (IFN- γ) (1). This immune activity leads to an overproduction of collagen by fibroblasts, resulting in thickened, hardened plaques in the affected areas of the skin. The classification system devised by Peterson et al. categorizes the disease according to clinical morphologic assessment into five groups: morphea en plaque (MEP), linear morphea (LM), generalized morphea (GM), deep morphea (DM), and bullous morphea (BM) (2). Morphea presents significant clinical challenges due to its complex pathophysiology and the limited efficacy of available treatment modalities.

Adipokines are cytokines secreted by adipocytes. They captured increasing research interest due to their crucial role in various signaling cascades, as well as their multidirectional impact on innate and acquired immunity. While previous studies revealed altered levels of adipokines in numerous inflammatory and fibrotic diseases, to date, no research has explored the possible role of adipokines in morphea (3, 4). Adiponectin is an adipokine that has been extensively studied for over thirty years due to its pleiotropic effects on various tissues and organs (5). The anti-inflammatory and antifibrotic influence of adiponectin makes it an interesting target of research concerning numerous disorders. In recent years, studies have shown that SSc is associated with significantly reduced adiponectin levels, suggesting its possible role in the development of both organ and skin fibrosis (6, 7). Notably, adiponectin exerts both pro-inflammatory and anti-inflammatory effects, showing different concentrations in various autoimmune and inflammatory diseases. Its levels are higher in patients with rheumatoid arthritis (RA) and systemic lupus erythematosus (SLE) but lower in patients with psoriasis (8–10). The precise mechanisms underlying these differences remain poorly understood, although the existence of adiponectin in three isoforms has been suggested to contribute to its multi-directional role in various diseases (11). However, there are no studies evaluating adiponectin levels in morphea.

To tackle this problem, we analyzed the concentration of adiponectin in the serum samples from patients diagnosed with different types of morphea, as well as explored its relationship with clinical parameters and severity of skin involvement. We hypothesized that adiponectin levels might be reduced in morphea patients compared to the control group, exhibit differences among different types of morphea, and correlate with disease duration and activity.

## 2 Materials and methods

### 2.1 Study population and study design

This study was a prospective, observational, single-center trial conducted at the Department of Dermatology, Poznan University of Medical Sciences in Poznan, Poland. Participants were prospectively enrolled between April 2018 and December 2019. The study involved patients diagnosed with morphea and treated in the Department of Dermatology at Poznan University of Medical Sciences in Poznan, Poland. The diagnosis of morphea was established based on the clinical presentation, ensuring that only clearly defined cases were included. All patients meeting the inclusion criteria were involved in this study. The clinical evaluation involved past medical history, the classification of morphea type according to Peterson et al., as well as the evaluation of the activity/intensity and tissue damage based on the localized scleroderma assessment tool (LoSCAT) (2). The LoSCAT encompasses two key components: the activity index (LoSAI) and the damage index (LoSDI). Additionally, it incorporates the Physician Global Assessment of both disease activity (PGA-A) and damage (PGA-D). The LoSCAT score sheet, adapted from Teske et al., may be found in the **Supplementary Material** (12). An active lesion is defined when a new erythematous lesion and/or sclerotic plaque appeared within the previous month, or if the spreading of pre-existing lesions or an erythematous halo (lilac ring) is observed (13, 14). The control group included healthy adults. In both study groups, subjects with infections, malignancy, and other autoimmune tissue diseases were excluded.

The study was approved by the ethics committee of Poznan University of Medical Sciences and informed consent was obtained from all study participants.

### 2.2 Sample collection and biomarker analysis

Whole blood samples were collected from patients and healthy controls and centrifuged at room temperature (RT) at 800 xg for 45 min (Eppendorf 5804R, Germany). Plasma samples were subsequently stored at -80°C until analysis for a maximum of 6 months (Revco). Serum adiponectin levels were measured using a BioTek Epoch microplate spectrophotometer (Agilent, US) and commercially available enzyme-linked immunosorbent assay kits (ELISA). Human Adiponectin ELISA Kit (EZHADP, Sigma-Adrich) was used following the manufacturer’s instructions.

### 2.3 Statistical analyses

The data was analyzed using the Paleontological Statistics (PAST) software (version 2.17c). The normal distribution of the continuous variables was evaluated using the Shapiro-Wilk test, along with normality tests based on Skewness and Kurtosis. As only some groups exhibited a normal distribution, a non-parametric Kruskal-Wallis test was used for the comparative analysis. The Spearman’s rank correlation coefficient was used to examine the relationship between adiponectin concentration and the LoSCAT score, as well as between adiponectin concentration and disease duration. The significance level was set at *p*<0.05.

## 3 Results

### 3.1 Demographic and clinical characteristic

A total of 67 eligible morphea patients and 30 healthy controls were recruited. **Table 1** shows the demographic and clinical parameters of the study participants.

**Table 1:** Demographic and clinical parameters in patients with morphea and the control group.

Characteristics	Morphea N = 67	Controls N = 30
Age, mean ± SD (range) years	44.7 ± 19.9 (9 – 81)	30.5 ± 4.0 (25 – 39)
Sex, n (%)		
Male	23 (34)	7 (23)
Female	44 (66)	23 (77)
Disease duration, mean ± SD (range) years	5.7 ± 8.0 (0.3 – 48.0)	NA
LoSCAT, median (range)	7 (2 – 68)	NA
Active disease, n (%)	41 (61)	NA
Morphea type, n (%)		
MEP	32 (48)	NA
GM	22 (33)	NA
LM	7 (10)	NA
DM	6 (9)	NA

SD, standard deviation; NA, not applicable; LoSCAT, localized scleroderma assessment tool; MEP, morphea en plaque; LM, linear morphea; GM, generalized morphea; DM, deep morphea.

### 3.2 Comparison of adiponectin concentrations

The analysis of serum adiponectin concentrations in groups of morphea patients and healthy controls showed significantly lower levels of adiponectin in morphea patients compared to healthy subjects (*p*=0.0005). **Table 2** depicts the adiponectin concentrations in both groups.

**Table 2:** Adiponectin concentrations in morphea patients, healthy subjects, and morphea subgroups (ng/ml).

	Morphea	Controls	MEP	LM	DM	GM	LM+DM+GM	active	non-active
N	67	30	32	7	6	22	35	41	26
Mean ± SD	6803.0 ± 3373.2	10111.7 ± 5531.7	6380.0 ± 3349.1	6871.4 ± 2598.3	8442.5 ± 4807.9	6949.2 ± 3271.9	7190.0 ± 3396.8	6903.6 ± 3629.7	6644.3 ± 2986.0
Median (min – max)	5816.2 (364.7 – 15333.2)	8532.7 (2869.9 – 24241.9)	5693.7 (364.7 – 15333.2)	7705.7 (1832.5 – 9204.0)	6107.2 (4502.6 – 14860.1)	5815.3 (721.0 – 14867.0)	6765.8 (721.0 – 14867.0)	5816.2 (364.7 – 14866.9)	5748.2 (2629.0 – 15333.2)

SD, standard deviation; MEP, morphea en plaque; LM, linear morphea; DM, deep morphea; GM, generalized morphea.

### 3.3 Differences in adiponectin concentrations among morphea types

We observed no significant differences in adiponectin concentrations among the various morphea types. There were also no significant alterations between patients with MEP and other variants of the disease (deep, generalized, or linear). However, compared to healthy controls, patients diagnosed with either MEP or GM had significantly lower serum adiponectin concentrations. Furthermore, there were significantly reduced levels of adiponectin in patients presenting with severe forms of the disease (the group included GM, DM, and LM) compared to healthy subjects. The results of the analysis are shown in **Tables 2**, **3**.

**Table 3:** Analysis of differences in adiponectin concentrations among morphea subgroups using the Kruskal-Wallis test.

	LM	DM	GM	LM+DM+GM	Controls
MEP	0.4314	0.3894	0.4030	0.2507	0.0042*
LM		0.9431	0.7792	0.8263	0.1939
DM			0.8011	0.8973	0.3842
GM				0.8633	0.0355*
LM+DM+GM					0.0266*

*Statistically significant, *p* < 0.05.

MEP, morphea en plaque; LM, linear morphea; DM, deep morphea; GM, generalized morphea.

Kruskal-Wallis result for the entire set is p=0.0678.

### 3.4 Adiponectin concentrations in active morphea compared to non-active phase

Next, we compared the serum adiponectin concentrations between the patients with active morphea and those in the non-active phase of the disease. The results of our analysis are shown in **Table 2**. We found no significant differences in adiponectin levels between the active and non-active phases of morphea (*p =* 0.7526).

### 3.5 Adiponectin concentrations and the LoSCAT score

The Spearman’s rank correlation coefficient between serum adiponectin concentrations and the LoSCAT score in morphea patients was 0.1411. However, we found no correlation between the LoSCAT score and adiponectin concentrations (*p* = 0.2780).

### 3.6 Adiponectin concentrations and disease duration

The Spearman’s rank correlation coefficient between serum adiponectin concentrations and the duration of the disease equaled 0.0818. In our group, there was no correlation between adiponectin levels and the duration of morphea in years (*p* = 0.5309).

## 4 Discussion

In our study, we demonstrated for the first time that serum adiponectin concentrations are significantly reduced in morphea patients compared to healthy controls. This is in line with our hypothesis based on previous studies, which evaluated the role of adiponectin in various inflammatory and fibrotic states. Patients diagnosed with SSc or liver fibrosis showed significantly lower adiponectin levels in the study groups compared to the control groups (6, 7, 15, 16). Further investigation into the mechanisms underlying this relationship could lead to innovative therapeutic strategies aimed at inhibiting fibrotic progression by normalizing adiponectin levels in affected individuals. Adiponectin concentrations are also lower in patients suffering from psoriasis and negatively correlate with the activity and duration of the disease (17, 18). Studies show that adiponectin exerts multiple positive effects on skin homeostasis, inhibiting the proliferation and differentiation of keratinocytes and suppressing the secretion of pro-inflammatory cytokines, including tumor necrosis factor α (TNF-α), interferon γ (IFN-γ), interleukin 6 (IL-6), IL-8, IL-17, and IL-22 (19–21). Contrastingly, it promotes the production of anti-inflammatory IL-1RA and IL-10 (22). Importantly, adiponectin inhibits skin fibroblast proliferation induced by tumor growth factor β1 (TGF-β1) *in vitro* (23). These findings suggest that adiponectin exerts protective effects against inflammation and fibrosis by modulating multiple signaling pathways. By modulating these signaling pathways, adiponectin plays a critical role in preserving cellular homeostasis and mitigating the pathological collagen accumulation linked to chronic inflammatory states. This underscores the potential of targeting adiponectin signaling as a therapeutic strategy in the management of conditions marked by inflammation and fibrosis. Our study shows that morphea patients present with lower serum levels of this protein, which may contribute to skin fibrosis typically observed in morphea, especially in the most severe forms, although we found no significant differences in adiponectin levels between patients with MEP, the most common and mildest type of morphea, and other variants of this disease, including more severe forms like deep, generalized, or linear. Nevertheless, we observed significantly reduced levels of circulating adiponectin in patients diagnosed with either MEP or GM compared to healthy individuals. In addition, the group including severe morphea variants (GM, DM, and LM) exhibited significantly lower adiponectin concentrations compared to the control group. These findings might be associated with the small number of patients presenting with either LM or DM. Therefore, further research is essential to understand the etiopathogenetic role of adiponectin in connection to other complex cytokine network interplay and explore its potential therapeutic implications in the management of morphea.

The absence of significant differences between the active and non-active phases of the disease raises questions about the role of adiponectin in disease dynamics. These results might suggest a minimal influence of adiponectin on the progression of morphea. Our findings are in line with previous studies on inflammatory diseases. In rheumatoid arthritis (RA) patients, there were no significant correlations between adiponectin levels and both clinical and laboratory markers of disease activity (24). However, a study on Japanese patients showed that adiponectin concentrations positively correlated with RA activity (25). Further studies should focus on assessing the correlation between levels of adiponectin and laboratory markers of inflammation in morphea, such as TGF-β, IL-1β, IL-2, IL-4, IL-6, IL-13, and IL-17A, among others (26–29). By elucidating how adiponectin interacts with these inflammatory markers, researchers may reveal significant implications for the management and treatment of this condition.

Our study revealed no correlations between adiponectin levels and either the duration of morphea in years or the severity of the process examined with the LoSCAT score. The LoSCAT includes the modified Localized Skin Severity Index (mLoSSI), a marker of disease activity, and the Localized Scleroderma Damage Index (LoSDI), measuring damage. Our findings also indicated no association between disease activity and adiponectin levels. Therefore, these results imply a questionable role of adiponectin in the progression of the disease.

The correlation between disease duration and adiponectin concentrations has already been explored in other autoimmune diseases. In contrast to our results, in RA patients, there was a positive correlation between serum adiponectin concentrations and the duration of the disease (24, 30). Interestingly, a similar positive correlation was found in patients suffering from SSc, as a disease duration of 7 years or longer was associated with significantly higher adiponectin levels (7). Since our study showed contrary results and no correlation between disease duration and adiponectin concentration, further research should take into account the plausible role of adiponectin in morphea pathogenesis but not in its clinical course and progression. Given that the previous research on SSc patients divided patients based on the duration of the disease into two groups, the lack of correlation in our patients might also result from a lack of creating two subgroups of either early or long duration of morphea (7). It should also be considered that in the course of SSc, fibrosis might affect not only the skin but also every internal organ. In contrast, morphea is limited to the skin and subcutaneous tissues. Therefore, disrupting the normal organ function in SSc may contribute to higher adiponectin levels as the disease continues.

## 5 Conclusions

Our study reports, for the first time, significantly lower levels of serum adiponectin in patients with morphea. However, adiponectin levels have no direct correlation with the severity or activity of the disease, suggesting that adiponectin may not be a reliable biomarker for clinical evaluation of the disease. Nevertheless, our findings underscore new avenues for investigation, highlighting the necessity for further research to explore the potential implications of adiponectin in the pathogenesis of morphea, particularly its role in inflammation and fibrosis. Understanding how adiponectin might contribute to the development of morphea may provide valuable insights for new therapeutic strategies in the future.

## Data Availability

The original contributions presented in the study are included in the article/**Supplementary Material**. Further inquiries can be directed to the corresponding author.
