# Supplementary material for: Is adiponectin involved in morphea pathogenesis? – first observational study
Source: Front Immunol. 2025 May 30;16:1588439. doi: 10.3389/fimmu.2025.1588439 (PMC12162899; doi:10.3389/fimmu.2025.1588439)
Supplement: Supplementary file 1 [file DataSheet1.pdf]

Supplementary material - Localized Scleroderma Cutaneous Assessment Tool (LoSCAT)

		Localized Scleroderma Skin Activity Index (LoSAI)			Localized Scleroderma Skin Damage Index (LoSDI)			
		New or Enlarged in past month	Erythema	Induration	Dermal atrophy	Subcutaneous atrophy	Hyper- or hypopigmentation	Skin thickness at center
		0 = none<br>3 = present	0 = none<br>1 = pink<br>2 = red<br>3 = dark red or violaceous	0 = none<br>1 = mild<br>2 = moderate<br>3 = marked	0 = none<br>1 = shiny<br>2 = visible vessels<br>3 = cliff drop	0 = none<br>1 = flat<br>2 = concave<br>3 = marked	0 = none<br>1 = mild<br>2 = moderate<br>3 = marked	0 = none<br>1 = mild<br>2 = moderate<br>3 = marked
	Scalp/Face							
	Neck							
	Chest							
	Abdomen							
	Upper back							
	Lower back							
Right	Arm							
	Forearm							
	Hand							
	Thigh							
	Leg							
	Foot							
Left	Arm							
	Forearm							
	Hand							
	Leg							
	Foot							

LoSAI = ___________ LoSDI = _________

Physician Global Assessment of Disease Activity (0 = inactive, 100 = markedly active) = ________

Physician Global Assessment of Disease Damage (0 = no damage, 100 = markedly damaged) = ________
